# Graphene and Other 2D Layered Nanomaterials and Hybrid Structures: Past, Present, and Future Directions

**DOI:** 10.3390/ma19102046

**Published:** 2026-05-14

**Authors:** Paolo Negro, Domenica Scarano, Federico Cesano

**Affiliations:** Chemistry Department and NIS (Nanomaterial for Industry and Sustainability) Interdepartmental Centre, University of Torino, and INSTM-UdR Torino, Via P. Giuria 7, 10125 Torino, Italy

## 1. From Graphene to a Broad 2D Materials Family

**Figure 1:**
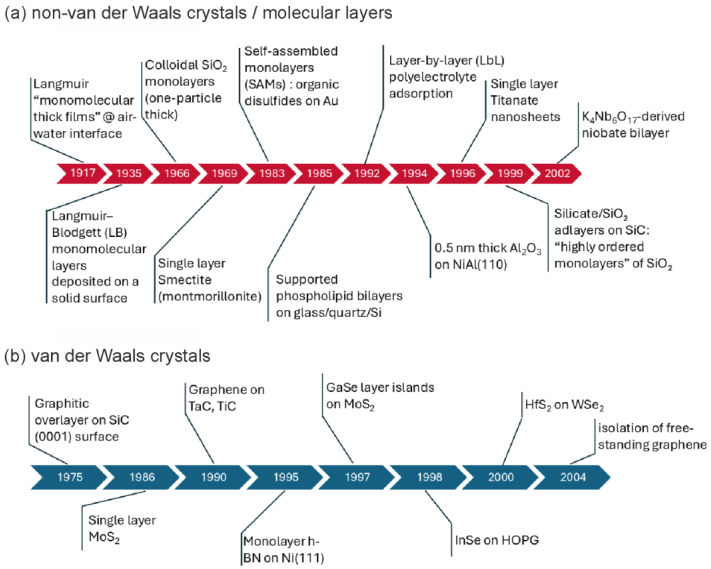
Historical timeline of 2D structures, showing the evolution of: (**a**) non-van der Waals and molecular layers and (**b**) van der Waals-layered materials.

Even before graphene was isolated (2004), many experiments established the existence of monomolecular layers at interfaces and the formation of transferable molecular monolayers on solids. These studies also produced ultrathin inorganic films (or molecular coverages) measuring a few atomic layers in thickness. Subsequent advances in high-resolution electron microscopy and scanning-probe methods, together with surface-sensitive spectroscopies, made it possible to directly measure and resolve the thickness, morphology, and in many cases, the atomic and electronic structure of 2D molecular assemblies, as well as ultrathin ionic, covalent, and van der Waals-layered crystals (Figure 1).

**Figure 2:**
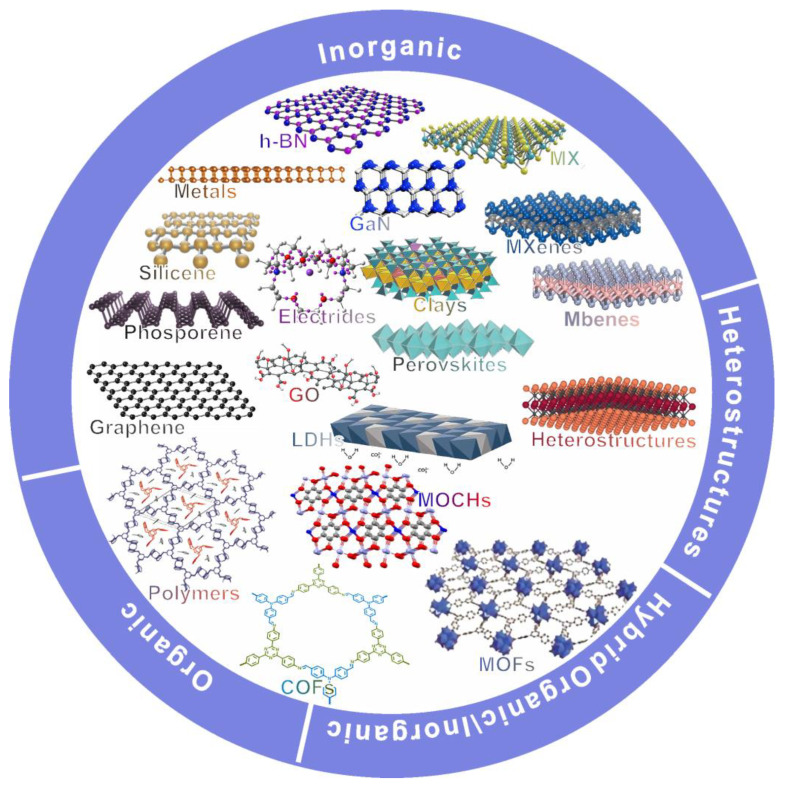
Schematic classification of two-dimensional materials and related hybrid structures.

After 2004, a rapid growth took place, moving from a few model systems to a broad set of 2D materials, including graphene, hexagonal BN, transition-metal dichalcogenides, and related layered oxides and chalcogenides. In a broader context, 2D materials now comprise carbon-based sheets, as well as inorganic compounds, including chalcogenides, nitrides, and carbides. The subject also covers 2D metal–organic frameworks (MOFs) and covalent organic frameworks (COFs), polymeric 2D sheets, and hybrid heterostructures [1], as shown in Figure 2. The atomic-scale thickness, the high anisotropy and the tuneable structure of these materials result in properties that are rarely found in traditional three-dimensional (3D) materials, i.e., high charge mobility, strong interactions with light and novel magnetic or ferroelectric behaviours. These materials are also distinguished by their low friction and distinctive interfacial chemistry.

## 2. Emerging 2D Materials (2022–2025): New Members of the Family

As highlighted in the previous Special Issue, the field of 2D materials has already moved well beyond its original graphene-centred boundaries [2]. Since 2022, a number of reports have led to significant advancements in the field of 2D materials research. In contrast to simply adding variants of known layered compounds, these studies have introduced new classes of atomically thin matter, including covalent fullerene sheets, elemental ferroelectric layers, monatomic metallic sheets, high-entropy 2D compounds, crystalline 2D polymers, and correlated 2D metal–organic frameworks.

A great development took place in the synthesis of molecular carbon-based 2D crystals from fused fullerene cages. Hou and colleagues [3] prepared a large-area, single-crystal monolayer quasi-hexagonal-phase fullerene (qHP-C60) through the interlayer-bond cleavage of a layered precursor, obtaining a 2D polymer in which each C60 cage is covalently connected in-plane to its neighbours with a honeycomb-like topology. This material combines thermodynamic stability and crystallinity with a moderate band gap, thereby addressing the zero-gap limitation of graphene and initiating multiple theoretical studies on its elastic, thermal and electronic properties.

Beyond carbon, Gou et al. [4] reported that a black-phosphorus-like monolayer of bismuth (BP-Bi) exhibits in-plane ferroelectricity, with spontaneous polarisation, that arises from ordered charge transfer and correlated lattice distortions between the two sublattices. Called “elemental ferroelectricity”, this behaviour challenges the conventional view that ferroelectricity requires specific ionic configurations or mixed-valence cations. BP-Bi therefore represents a promising platform for ultrathin non-volatile memories and for coupling polar order to spin, valley, and topological degrees of freedom.

Another notable development was the reported synthesis of atomically thin gold sheets derived from a non-van der Waals precursor. Kashiwaya et al. [5] used MAX-phase chemistry to prepare Ti_3_AuC_2_ by substituting Au into the layered ternary carbide, followed by controlled wet-chemical etching of the Ti–C slabs to obtain monolayer Au sheets. The resulting material exhibited a marked in-plane lattice shrinkage relative to bulk Au and a shift in the Au 4f binding energy, indicating a modified electronic structure in the two-dimensional domain. Although ab initio calculations suggest that an ideal Au monolayer is only metastable, the experiments indicated that curling and agglomeration effects could be mitigated through appropriate processing and surfactant-assisted stabilisation.

A third emerging theme is the rise in compositionally complex, high-entropy (HE) 2D materials. As demonstrated by Qu et al. [6], a hexanary 2D transition-metal dichalcogenide, (MoWReMnCr)S_2_, can be synthesised by a relatively low-temperature route and employed as an efficient hydrogen-evolution electrocatalyst. The material exhibited a layered framework analogous to MoS_2_, yet with a distinctive distribution of multiple metals across the cation sublattice. This distribution introduces local lattice distortions and a broadened electronic landscape, collectively contributing to enhanced catalytic behaviour. As for the fundamental science, the entropic stabilisation of nanosheets represents a potentially effective approach to the production of 2D materials, which otherwise cannot be produced due to their inherent instability in the low-dimensional limit.

Furthermore, crystalline two-dimensional polymers (2DPs) and covalent organic frameworks (COFs) have undergone a qualitative leap. In their report, Wang and colleagues [7] described the on-water surface synthesis of charged 2D polymer single crystals via an irreversible Katritzky reaction, through an irreversible reaction assisted by a surfactant monolayer. This process produces centimetre-scale, few-layer crystalline sheets that function as anion-selective membranes and electrode skin as functional materials for nanofluidics, energy conversion, and protective layers in energy devices.

Alongside these major advances in synthesis, the discovery of two-dimensional materials is increasingly being boosted by computational approaches, which shifts the field from empirical trial and error to predictive, data-driven approaches. From this perspective, high-throughput density functional theory remains essential for identifying layered precursors, evaluating exfoliation thermodynamics, assessing dynamical stability and screening electronic properties. Meanwhile, graph-based and topology-informed structural analyses are increasingly being used to detect hidden, low-dimensional motifs within chemically complex crystals. Machine-learning methods are also accelerating this discovery process by identifying structure–property relationships, optimising synthetic processes, facilitating inverse materials design and, more recently, autonomously improving growth and exfoliation protocols. Within this broader research landscape, a recent study by Björk et al. [8] represented a significant advance in extending computational discovery beyond van der Waals solids and the conventional MXene precursor space. Through a combination of topological screening, chemically resolved exfoliation thermodynamics, vacancy formation energetics and phonon-based stability analysis, the authors identified 119 chemically exfoliable layered precursors corresponding to 42 distinct, dynamically stable 2D materials. A more recent study by Zhang et al. [9] introduced a topological decoding strategy based on modularity for strongly bonded non-van der Waals crystals. This strategy demonstrates that conventional distance-based algorithms systematically overlook a significant proportion of embedded two-dimensional structural units. Through the selective removal of specific elements to create artificial substructures, followed by layer-recognition analysis, the authors identified 8889 hidden 2D motifs in ternary compounds. They demonstrated that the majority of these motifs would be missed by standard screening approaches and further isolated 1083 ternary oxides as thermodynamically promising candidates for proton-exchange exfoliation.

## 3. Contributions to This Special Issue

In the broad and dynamic landscape of 2D materials, the five papers contained in this Special Issue demonstrate how layered and two-dimensional materials can be used for environmental remediation, critical element recovery, photovoltaic energy conversion, interfacial physics, and photocatalysis.

Alazreg et al. [10] explores the “memory effect” of magnesium-aluminium layered double hydroxides (LDHs) and their calcined derivatives as a strategy for controlled fluoride uptake and release. MgAl-LDH is synthesised via the conventional coprecipitation method using nitrate precursors. This yields a homogeneous layered structure that transforms into a mixed MgAl layered double oxide (LDO) upon calcination at 450 °C. This thermally induced collapse of the hydroxide galleries produces an oxide phase that can subsequently reconstruct its layered architecture when brought into contact with aqueous solutions containing anionic species. When MgAl-LDO is rehydrated in water containing fluoride, the original LDH structure regenerates and fluoride anions are selectively inserted into the interlayer galleries to balance the positive charge of the brucite-like sheets. Elemental analysis and EDS measurements demonstrate that this reconstruction process results in significantly higher fluoride content.

The paper by Knapik et al. [11] addresses the increasingly important issue of recovering lithium from unconventional aqueous resources by presenting a new type of hybrid 2D adsorbent. This adsorbent combines graphene oxide with crown ether receptors within a polymeric hydrogel framework. The work begins with the synthesis of graphene oxide functionalised with two different crown ether motifs, 2-hydroxymethyl-12-crown-4 and hydroxy-dibenzo-14-crown-4, using epichlorohydrin as a bifunctional linker to covalently anchor the macrocycles onto the oxidised carbon lattice. These crown ether-grafted graphene oxide (GO) materials are then embedded in chitosan–poly(vinyl alcohol) hydrogels to produce mechanically robust composites with a high density of potential lithium (Li^+^) binding sites distributed between the carboxylated graphene matrix and the macrocyclic cavities. Extensive characterisations using FTIR, XRD, Raman spectroscopy and SEM–EDS confirm the successful grafting of the crown ethers, the preservation of the layered carbon framework, and the homogeneous distribution of the functional phase within the composite.

The contribution by Mehrabian et al. [12] focuses on designing and numerically optimising environmentally friendly perovskite solar cell architectures. These architectures replace the lead-based MAPbI_3_ absorber with formamidinium Tin iodide (FASnI_3_) and combine it with two-dimensional ZnO and Cu_2_O nanolayers that serve as electron and hole transport materials, respectively. The study uses a SCAPS-1D simulation framework and begins by calibrating the model against a reference experimental device based on an FTO/ZnO/MAPbI_3_/Cu_2_O/Au stack. Through the precise adjustment of material parameters, defect densities, and interface properties, the author achieves a conversion efficiency of 6.06%, with an open-circuit voltage of 0.76 V, a short-circuit current density of 12.26 mA/cm^2^, and a fill factor approaching 65%. These results are in line with those reported in the literature. This validation step is crucial because it establishes the reliability of the simulation approach and enables meaningful extrapolation to the Pb-free configuration.

Mikšić Trontl et al. [13] reported high-resolution scanning tunnelling microscopy to examine the initial stages of gold intercalation in graphene grown on Ir(111). This study provides microscopic insight into how a two-dimensional overlayer modifies the growth and reconstruction dynamics of metals at buried interfaces. Starting with a graphene/Ir(111) system, sub-monolayer amounts of gold are deposited and then driven to intercalate at the interface between the carbon sheet and metallic substrate. STM imaging reveals that the morphology of the emerging gold islands under graphene differs markedly from that observed when gold is grown directly on bare Ir(111). While gold typically forms ramified, dendritic islands on the uncovered Ir surface, which are characteristic of diffusion-limited aggregation under restricted adatom mobility, the presence of graphene increases Au adatom mobility. This results in the formation of compact, isotropic islands in the intercalated configuration. This reflects a significant modification to the surface diffusion barriers and nucleation energetics caused by the graphene cover. This can be understood as a flexible yet confining ‘ceiling’ that alters the potential landscape experienced by gold adatoms.

Negro et al. [14] presented a detailed, multifaceted study of graphitic carbon nitride obtained via the thermal polymerisation of melamine. They combine advanced experimental characterisation with dispersion-corrected density functional theory to clarify the structural, optical, and vibrational properties of this significant metal-free photocatalyst. The authors prepare the polymerised carbon nitride through the straightforward calcination of melamine under mild conditions, then subject the resulting material to X-ray powder diffraction, scanning electron microscopy, atomic force microscopy, ultraviolet–visible spectroscopy and Fourier-transform infrared spectroscopy (FTIR) analysis. These techniques reveal the expected layered structure of g-C_3_N_4_, with lamellar stacking in one crystallographic direction and a relatively low surface area, as well as morphological features consistent with aggregated platelets and sheets. UV–vis data show an absorption onset in the visible region, corresponding to an indirect bandgap of approximately 2.59 eV. Additionally, the FTIR spectrum exhibits a complex pattern of bands associated with heptazine ring breathing modes, C–N stretching and NH and NH_2_ groups, as well as out-of-plane deformations.

## References

[1] Muñoz J. (2024). Rational Design of Stimuli-Responsive Inorganic 2D Materials via Molecular Engineering: Toward Molecule-Programmable Nanoelectronics. Adv. Mater..

[2] Scarano D., Cesano F. (2021). Graphene and Other 2D Layered Nanomaterials and Hybrid Structures: Synthesis, Properties and Applications. Materials.

[3] Hou L., Cui X., Guan B., Wang S., Li R., Liu Y., Zhu D., Zheng J. (2022). Synthesis of a monolayer fullerene network. Nature.

[4] Gou J., Bai H., Zhang X., Huang Y.L., Duan S., Ariando A., Yang S.A., Chen L., Lu Y., Wee A.T.S. (2023). Two-dimensional ferroelectricity in a single-element bismuth monolayer. Nature.

[5] Kashiwaya S., Shi Y., Lu J., Sangiovanni D.G., Greczynski G., Magnuson M., Andersson M., Rosen J., Hultman L. (2024). Synthesis of goldene comprising single-atom layer gold. Nat. Synth..

[6] Qu J., Elgendy A., Cai R., Buckingham M.A., Papaderakis A.A., de Latour H., Hazeldine K., Whitehead G.F.S., Alam F., Smith C.T. (2023). A Low-Temperature Synthetic Route Toward a High-Entropy 2D Hexernary Transition Metal Dichalcogenide for Hydrogen Evolution Electrocatalysis. Adv. Sci..

[7] Wang Z., Zhang Z., Qi H., Ortega-Guerrero A., Wang L., Xu K., Wang M., Park S., Hennersdorf F., Dianat A. (2022). On-water surface synthesis of charged two-dimensional polymer single crystals via the irreversible Katritzky reaction. Nat. Synth..

[8] Björk J., Zhou J., Persson P.O.Å., Rosen J. (2024). Two-dimensional materials by large-scale computations and chemical exfoliation of layered solids. Science.

[9] Zhang M., Ni X., Wang L., Luo J., Chen H., Zou X. (2025). Beyond van der Waals: Modular Identification of 2D Materials in Strongly Bonded Layered Crystals. Angew. Chem. Int. Ed..

[10] Alazreg A., Tadić V., Egelja A., Savić A., Šaponjić A., Vuksanović M.M., Heinemann R.J. (2025). Memory Effect of Double Oxides Compared to Simple Ion Exchange for Controlled Fluoride Ion Capture and Release. Materials.

[11] Knapik E., Rotko G., Piotrowski M., Marszałek M. (2024). Crown Ether-Grafted Graphene Oxide-Based Materials—Synthesis, Characterization and Study of Lithium Adsorption from Complex Brine. Materials.

[12] Mehrabian M., Taleb-Abbasi M., Akhavan O. (2024). Using Cu_2_O/ZnO as Two-Dimensional Hole/Electron Transport Nanolayers in Unleaded FASnI_3_ Perovskite Solar Cells. Materials.

[13] Mikšić Trontl V., Jedovnicki I., Pervan P. (2023). STM Study of the Initial Stage of Gold Intercalation of Graphene on Ir(111). Materials.

[14] Negro P., Cesano F., Casassa S., Scarano D. (2023). Combined DFT-D3 Computational and Experimental Studies on g-C_3_N_4_: New Insight into Structure, Optical, and Vibrational Properties. Materials.

